# A solid oxide photoelectrochemical cell with UV light-driven oxygen storage in mixed conducting electrodes

**DOI:** 10.1039/c6ta08110j

**Published:** 2016-12-12

**Authors:** Gregor Walch, Bernhard Rotter, Georg Christoph Brunauer, Esmaeil Esmaeili, Alexander Karl Opitz, Markus Kubicek, Johann Summhammer, Karl Ponweiser, Jürgen Fleig

**Affiliations:** a Institute of Chemical Technologies and Analytics , TU Wien , Vienna , Austria . Email: j.fleig@tuwien.ac.at; b Institute for Energy Systems and Thermodynamics , TU Wien , Vienna , Austria; c Institute of Atomic and Subatomic Physics , TU Wien , Vienna , Austria

## Abstract

A single crystalline SrTiO_3_ working electrode in a zirconia-based solid oxide electrochemical cell is illuminated by UV light at temperatures of 360–460 °C. In addition to photovoltaic effects, this leads to the build-up of a battery-type voltage up to more than 300 mV. After switching off UV light, this voltage only slowly decays. It is caused by UV-induced oxygen incorporation into the mixed conducting working electrode and thus by changes of the oxygen stoichiometry *δ* in SrTiO_3–*δ*_ under UV illumination. These changes of the oxygen content could be followed in time-dependent voltage measurements and also manifest themselves in time-dependent resistance changes during and after UV illumination. Discharge currents measured after UV illumination reveal that a large fraction of the existing oxygen vacancies in SrTiO_3_ become filled under UV light. Additional measurements on cells with TiO_2_ thin film electrodes show the broader applicability of this novel approach for transforming light into chemical energy and thus the feasibility of solid oxide photoelectrochemical cells (SOPECs) in general and of a “light-charged oxygen battery” in particular.

## Abstract 2

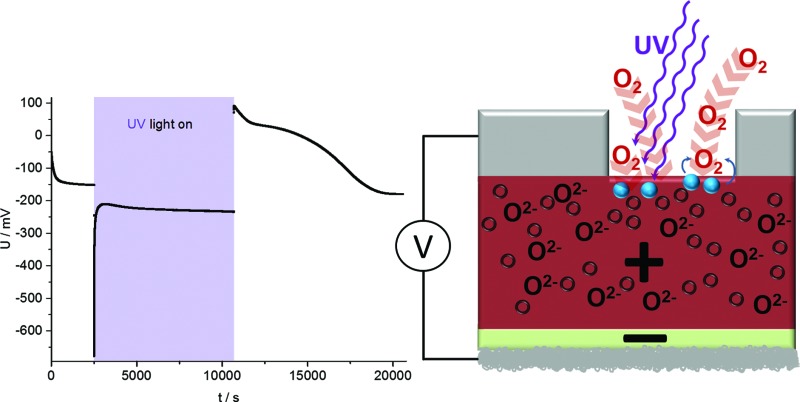
A SrTiO_3_ working electrode at 360–460 °C incorporates oxygen under UV illumination. This leads to a voltage in a solid oxide (photo-)electrochemical cell..

## 1. Introduction

Satisfying the growing energy demand in the light of multiple boundary conditions such as climate change or the strive for autarchy from fossil fuels has drawn much attention to renewable energy sources. Despite continuing efforts throughout the fields of chemistry, physics and materials science, efficiently harvesting and storing the sun's energy remains a big challenge. This situation causes the need to identify and investigate new light conversion and storage systems. An important approach is based on light as a driving force for an electrochemical reaction. The reaction products, for example H_2_ and O_2_ from the splitting of water, can then be stored. Photoelectrochemical systems using aqueous electrolytes have been intensively studied for decades,^1–7^ with TiO_2_, Fe_2_O_3_ and other transition metal oxides being the favoured materials for photo(electro-)chemical water splitting.^5,6,8^ However, the effect of light on cells based on inorganic solid electrolytes, for example oxide ion conductors, has been hardly investigated so far. This is true despite the detailed knowledge available on the electrochemical properties of oxide ion conductors and mixed conductors used, for example, in solid oxide fuel and electrolysis cells.

In a recent publication we have shown that combination of an oxide-based high temperature solar cell and a solid oxide electrochemical cell allows oxygen pumping from low to high pressure; the corresponding photovoltaic cell delivers more than 900 mV open circuit voltage at 400 °C.^9^ However, a solid electrolyte based photoelectrochemical cell with a photoactive electrode working at high temperatures has not been presented so far. Among the scarce work relevant for potential solid oxide photoelectrochemical devices are the theoretical study by Ye *et al.*,^10^ the work on anodic TiO_2_ thin films by Gundlach & Heusler^11^ and the investigation on SrTiO_3_ single crystals by Merkle *et al.* ^12^ In the latter two studies, it was found that after jumps in oxygen partial pressure at high temperatures both SrTiO_3_ and TiO_2_ incorporate oxygen faster under UV light. However, it has not been investigated in depth whether UV light mainly affects the reaction kinetics or also strongly changes the oxygen stoichiometry of the materials, *i.e.* alters thermodynamic properties and thus enables the build-up of thermodynamic driving forces.

In the present study, we show that operating solid oxide photoelectrochemical cells (SOPECs) can be realised by using large band gap mixed conducting oxides as photoactive electrodes on yttria-stabilized zirconia electrolytes. Single crystalline SrTiO_3_ electrodes, but also TiO_2_ thin film electrodes were exposed to UV light at temperatures in the range of 360 °C to 460 °C. This lead to the build-up of a Nernst voltage in the 100 to 300 mV range, which decreased only slowly after UV exposure. Numerous additional experiments including measurements of conductivity and discharge current as well as variations of UV exposure time and current collector material allowed us to suggest a consistent explanation of the observed phenomena: upon UV light the mixed conducting oxide electrodes incorporate additional oxygen and thus exhibit a higher oxygen chemical potential than before UV exposure. Accordingly, the solid oxide electrochemical cell becomes “charged” by UV light, in analogy to a battery charged by an electric current.

## 2. Experimental

### 2.1. SrTiO_3_-based SOPECs

**Fig. 1:**
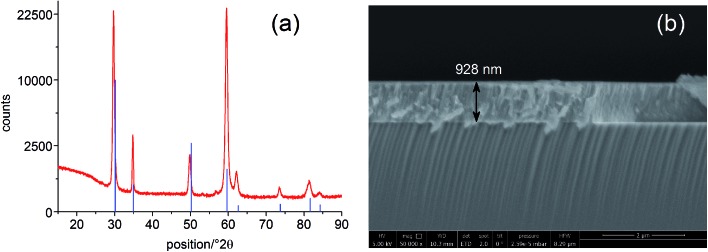
(a) Grazing incidence X-ray diffraction pattern of a YSZ thin film deposited on STO by PLD. Comparison with the database pattern (PDF 04-018-5452, 6 mol% YSZ, blue lines) confirms the successful deposition of YSZ. (b) SEM image (cross section) of the YSZ thin film electrolyte on the STO single crystal.

The solid oxide photoelectrochemical cells (SOPECs) used in this study include a mixed conducting electrode with current collector and an oxide ion conducting electrolyte. SOPECs based on SrTiO_3_ (STO) were prepared from nominally undoped STO single crystals (5 × 5 × 0.5 mm^3^ and 10 × 10 × 0.5 mm^3^, Crystec GmbH, Germany). Yttria-stabilized zirconia (YSZ) thin film electrolytes of about 900 nm thickness were deposited on these STO substrates by pulsed laser deposition (PLD; Kr/F excimer laser Lambda COMPex Pro 205F, wavelength = 248 nm, 10 Hz, 400 mJ per pulse, 60 min deposition time) while keeping the samples at about 650 °C (4 cm distance from the target, 4 × 10^–2^ mbar oxygen). The target for the YSZ electrolyte deposition was prepared from 8 mol% YSZ (Tosoh, Japan), sintered at 1200 °C.^13^ An as-deposited YSZ film was characterised by grazing incidence X-ray diffraction (XRD) on a PANalytical Empyrean diffractometer (2° incident angle, Cu K_α_ radiation). Comparison of the diffractogram with literature data from the ICDD PDF-4+ 2014 database^14^ confirmed the successful deposition of a phase-pure YSZ thin film, see Fig. 1(a). A cross section of the film measured by scanning electron microscopy (SEM; FEI Quanta 200 FEG) is shown in Fig. 1(b).

**Fig. 2:**
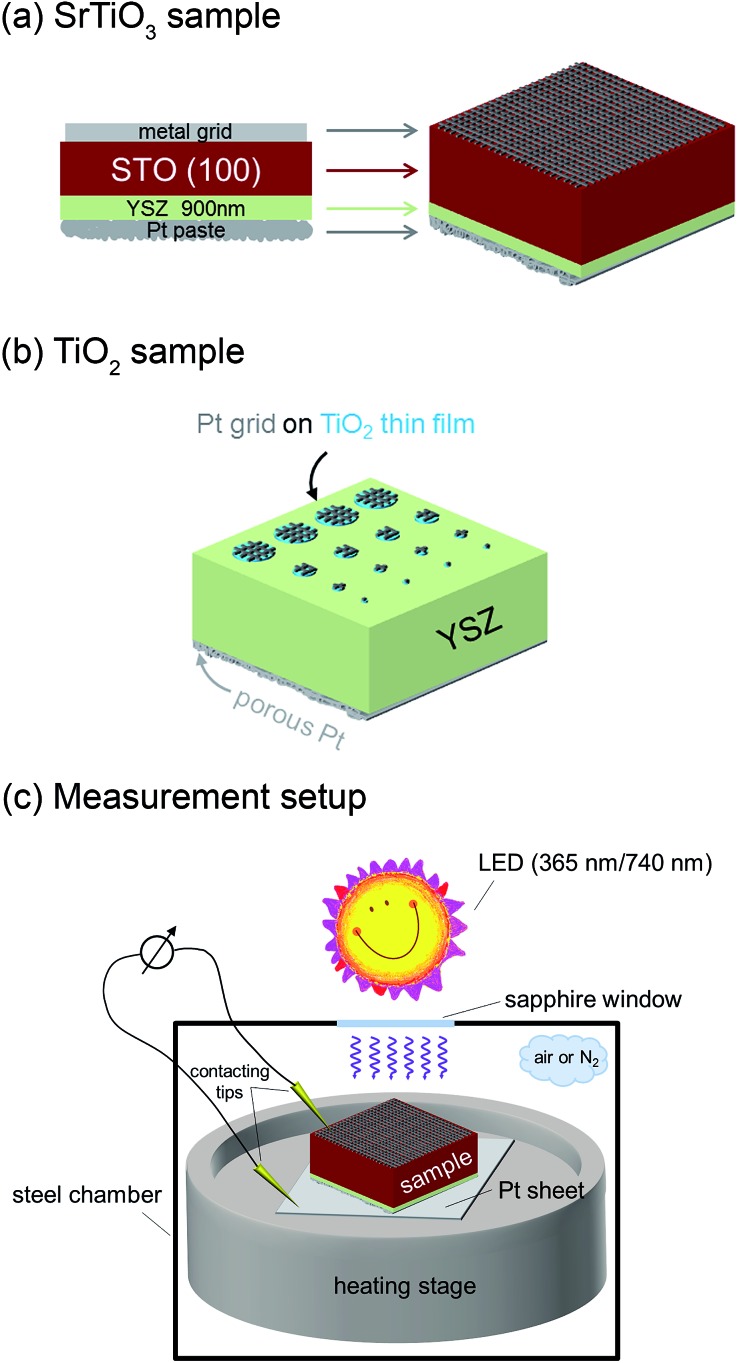
Sketches of a STO based SOPEC with YSZ thin film electrolyte (a), a TiO_2_ based SOPEC on a YSZ single crystal (b) and the measurement setup (c).

A grid-like thin film current collector was deposited on the STO electrode (on the opposite side of YSZ). For this, a Pt or Au thin film (≈100 nm) was produced by DC magnetron sputtering in Ar (BAL-TEC MED 020 Coating System; pressures and currents for Pt were 2 × 10^–2^ mbar and 100 mA, for Au 1 × 10^–2^ mbar and 50 mA) and subsequently micro-structured into a grid (stripes of 15 μm with 50 μm period) by photolithography and ion beam etching. A porous Pt counter electrode was applied onto the YSZ thin film electrolyte by brushing Pt paste obtained from Gwent Group, UK. Hence, an electrochemical cell with a STO single crystal as working electrode resulted. A sketch of the complete SOPEC is shown in Fig. 2(a). Two series of such STO-based cells were investigated. Series 1 included two samples, produced from two different 5 × 5 mm^2^ STO single crystals of the same batch. Series 2 consisted of four samples resulting by breaking apart a 10 × 10 mm^2^ STO single crystal of another batch. For comparison, also STO samples without YSZ layer were prepared and investigated.

### 2.2. TiO_2_-based SOPECs

Single crystalline (100) YSZ substrates (10 × 10 × 0.5 mm^3^ and 5 × 5 × 0.5 mm^3^; 9.5 mol% Y_2_O_3_, Crystec GmbH, Germany) were used as electrolytes for SOPECs with titania electrodes. Here, a Ti thin film with a nominal thickness of 100 nm was deposited by DC magnetron sputtering in Ar (pressure = 7 × 10^–3^ mbar, current = 100 mA). It was subsequently annealed for 2 hours at 550 or 900 °C in air, giving TiO_2_ layers with a thickness between 110 and 130 nm (measured by SEM as described above). The crystal structure was determined by grazing incidence XRD (see above) and the anatase phase could be identified. Grid-like, approximately 100 nm thick Pt current collectors were deposited on the titania thin films by sputtering and lift-off photolithography (see above). The resulting electrodes were micro-structured into circular working electrodes of a few 100 μm diameter by standard photolithography followed by Ar ion beam etching. The electrochemical cell was completed by a porous Pt counter electrode applied in the same way as for the STO samples. Fig. 2(b) shows a sketch of a complete TiO_2_-based SOPEC.

### 2.3. Measurement setup

SOPEC samples were placed on a Pt sheet and put on a microscope heating stage (Linkam, UK). In order to allow illumination, the working electrodes were only locally contacted with acupuncture needles (Wujiang City Cloud & Dragon; Model “Diamant”, Karl Blum, Germany; Model “EGON”, schwa-medico, Germany), the counter electrodes were contacted by the Pt sheet. The whole arrangement (heating stage and contacting tools) was placed in a stainless steel chamber with a sapphire window above the sample. Most measurements were carried out in ambient air, some data are also given for nitrogen with a typical oxygen content in the ppm range. Set temperatures of the heating stage were generally 400 °C or 500 °C. Owing to asymmetric heating the true sample temperature was about 20–40 °C lower.^15,16^ In the following, all temperatures refer to estimated true sample temperatures, *i.e.* 360 and 460 °C, instead of 400 and 500 °C set temperature, respectively.

Impedance measurements were performed using Alpha-A impedance analysers by Novocontrol, Germany, either with ZG-2, ZG-4 or POT/GAL interfaces. For fast impedance measurements a HP 4192A was employed. For voltage measurements digital multimeters were used (model 2000 and model 616, Keithley, USA) and current measurements were carried out on a Keithley 6487 picoammeter.

**Fig. 3:**
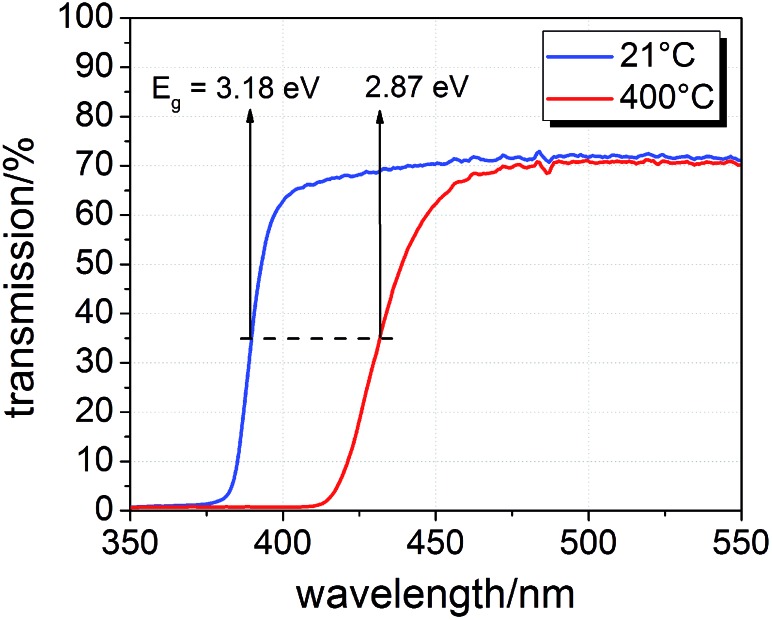
Transmission spectra of a SrTiO_3_ single crystal (0.5 mm thickness) at room temperature and 400 °C in air with band gap energies *E*_g_ estimated from half maximum transmission.

The samples were illuminated by light emitting diodes (LEDs) mounted about 2.4 cm above the sample. A sketch of the entire set-up is shown in Fig. 2(c). Two types of LEDs from Led Engin Inc., USA (LZ4-00U600, 365 nm, 11 W electric power; LZ4-00R300, 740 nm, 6.3 W electric power) were employed. The intensity at the sample surface was estimated to about 6–9 mW cm^–2^ for the UV LED. Values for the red LED were about 12–18 mW cm^–2^. UV photons emitted by the 365 nm LED have an energy of about 3.4 eV and can therefore excite electrons across the band gaps of STO and TiO_2_, which are both approximately 3.2 eV (ref. 9 and 17) at room temperature and even lower at higher temperatures.^18^ This is also illustrated by transmission spectra of STO single crystals measured at room temperature and 400 °C in air (Fig. 3). Red light does not lead to additional electron/hole generation.

## 3. Voltage measurements in SOPECs: general features and interpretation

### 3.1. Voltage measurements under UV light

**Fig. 4:**
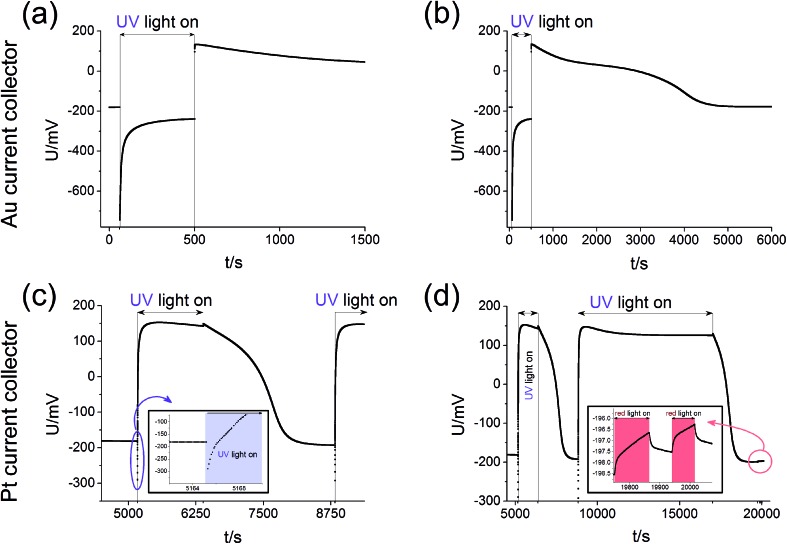
Typical *U*(*t*) curves, measured on STO samples with YSZ electrolyte (series one) at 360 °C in air with Au current collector (a and b) or Pt current collector (c and d). (b) and (d) display experiments on a longer time scale with complete relaxation for the sample with Au current collectors (b) and repeated illumination of the sample with Pt current collectors in (d). Magnifications of special parts of (c) and (d) indicate the short time changes upon UV light (c) and the only very tiny effect of red light (d).

Measurements of the open circuit voltage *U* of STO based solid oxide photoelectrochemical cells (SOPECs) were carried out as a function of time *t* while switching UV light on and off. Typical *U*(*t*) curves resulting from such measurements are shown in Fig. 4 for Au and Pt current collectors. A voltage offset was present even without illumination, it reached an acceptably stable value after some time (before illumination). Upon switching on UV light, a fast jump to a more negative voltage occurred. Afterwards, the voltage increased and in the case shown in Fig. 4(c) and (d) it even went far beyond the initial voltage. Eventually, a kind of plateau was reached. Upon switching UV light off, a sudden jump to a more positive voltage occurred (only a tiny spike in Fig. 4(c) and (d)). The voltage then returned to its initial value within a few thousand seconds. This behaviour was reproducible, see Fig. 4(d).

Fig. 4(c) also shows a zoom to the moment when UV light was switched on; the quick jump to more negative voltages and the subsequent increase to less negative voltages can be clearly seen. In panel (d) another magnification shows the effect of red light. Upon switching on and off red light only a very small change in voltage resulted (about 1–2 mV) compared to the effect of UV light (up to several 100 mV). This small voltage effect of red light is attributed to heating; accordingly all larger voltage effects found for UV illumination are not caused by thermal effects such as thermovoltages.

**Fig. 5:**
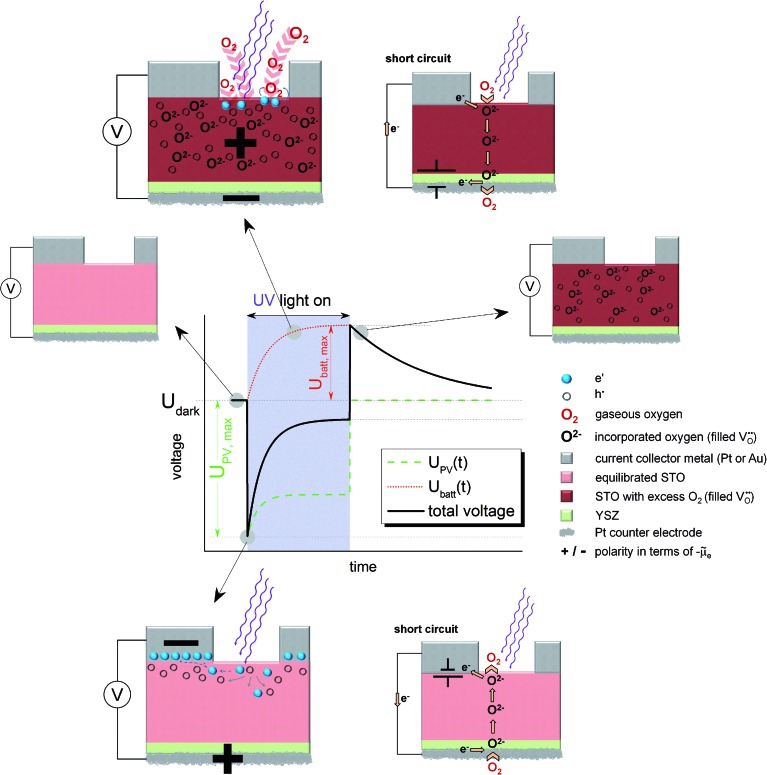
Schematic *U*(*t*) curves detailing the contributions from the battery voltage, *U*_batt_, and the photovoltaic voltage, *U*_PV_, which add up to the total voltage. The sketches indicate the states before and after UV illumination, the photovoltaic processes under UV light (bottom, open circuit refers to the situation indicated in the *U*(*t*) curve) and the battery type processes under UV light (top, open circuit refers to the red *U*_batt_ curve). Polarities refer to the electrochemical potential of the electrons (*μ̃*_e_). In both cases also a short circuited cell with oxygen pumping is sketched; the location of the corresponding driving force is indicated by a battery symbol.

For the sake of clarity, we first present our model to explain the measured *U*(*t*) curves and then provide numerous additional experimental data to support the validity of this interpretation (Section 4). The model is based on the schematic *U*(*t*) curve in Fig. 5 which reflects the main phenomena found in the experiments on SOPECs with STO working electrodes. This figure illustrates that UV illumination leads to two types of voltages, which both exhibit a certain time dependence. One voltage is caused by a photovoltaic process (*U*_PV_, green in Fig. 5) and the other one is of electrochemical nature (*U*_batt_, red in Fig. 5). The maximum photovoltaic voltage *U*_PV,max_ is determined from the distance between the most negative point in the *U*(*t*) curve and the dark value *U*_dark_; it is thus found right after switching on UV light. The maximum battery-type voltage *U*_batt,max_ equals the distance between the most positive point in the *U*(*t*) curve and *U*_dark_; it is found shortly after switching off UV light.

### 3.2. The battery voltage

The existence of a battery-type electrochemical voltage *U*_batt_ and its time dependence can be understood as follows: within the absorption zone UV light generates electron–hole pairs in STO. This leads to a splitting of the Fermi level into two quasi Fermi levels and in particular the minority charge carrier concentration strongly changes. Nominally undoped STO is hole conducting in most cases, with electrons being minority charge carriers.^19,20^ Thus, UV light has the largest relative effect on the concentration of electrons. A chemical consequence of this electron concentration enhancement was already described in ref. 12 and refers to the kinetics of oxygen reduction at the STO surface: at temperatures of a few hundred degrees, a permanent oxygen exchange takes place between STO and the gas phase according to the surface reaction ½O_2_ + 2e′ ⇔ O^2–^.^12,16,18,21^ Under UV illumination, the forward reaction of this oxygen exchange, *i.e.* oxygen reduction, becomes strongly accelerated, most probably due to conduction band electrons participating in the rate-determining reaction step of the corresponding reaction.^12,22^

This enhancement of the oxygen reduction rate under illumination leads to a non-equilibrium situation and a net oxygen uptake of STO under UV light. Accordingly, a change in the oxygen non-stoichiometry *δ* of SrTiO_3–*δ*_ results, see sketches in Fig. 5. Neither a current collector nor an electrolyte is needed in this photochemical oxygen uptake. Its rate is determined by the oxygen chemical diffusivity in STO and the rates of oxygen reduction and release under UV illumination. The kinetics of the surface reactions may be influenced by a surface space charge layer. However, since not only electrons but also oxygen vacancies are required for oxygen reduction, and both are affected by space charges, it is difficult to predict how such a space charge affects the reaction kinetics. A simple interpretation of space charge effects in terms of electronic charge carrier separation might be misleading.

The oxygen stoichiometry change originates at the surface but oxygen diffusion in STO leads to its broadening even beyond the absorption depth of UV light and the diffusion length of photogenerated charge carriers. Essentially, the entire STO single crystal becomes affected by this stoichiometry change, see sketch in the top part of Fig. 5. In our case, the illuminated mixed conducting STO is used as an electrode of an electrochemical cell with oxide ion conducting electrolyte. Therefore, the stoichiometry changes become observable as a Nernst-type voltage between the two sides of the cell. This electrochemical voltage is determined by the new oxygen concentration in the working electrode under UV light and the oxygen chemical potential at the counter electrode, which is still in equilibrium with the surrounding gas. Fig. 5 displays the build-up of this “battery voltage” *U*_batt_ due to net oxygen uptake in STO as a dotted red line. *U*_batt,max_ values up to more than 300 mV are found at 360 °C in air with a positive polarity at the STO electrode, in accordance with its increased oxygen chemical potential. This also means that our SOPEC becomes chemically charged under UV illumination, similar to the electrical charging of a Li ion battery by intercalating Li into carbon.

An external short-circuit of an illuminated cell with only this *U*_batt_ being relevant would lead to a continuous oxygen pumping from the gas around the STO electrode to the gas at the counter electrode, see sketch in the top part of Fig. 5. Please note that in such an operating SOPEC the electrochemical reaction takes place at the gas/semiconductor (STO) interface, in contrast to a conventional water based photoelectrochemical cell with the electrochemical reaction being at the semiconductor/electrolyte interface. (A similar difference exists between solid oxide fuel cell electrodes and electrodes in fuel cells with aqueous electrolytes.^23^) Separation of light-induced charges at the metal/semiconductor or semiconductor/electrolyte interfaces is thus not essential in this type of photoelectrochemical cell. Right after switching off UV light, this battery-type voltage is still present (see sketch in Fig. 5). However, in the dark the STO crystal again releases its excess oxygen and thus the battery voltage decreases with time. This decrease is slower than the voltage increase under UV illumination, since the reaction rate of the oxygen release reaction is slower than that of the incorporation under UV light.

### 3.3. The photovoltaic voltage

The second UV-induced voltage in the SOPEC is photovoltaic in nature and is indicated in the sketches at the bottom of Fig. 5. This voltage simply reflects the fact that at most semiconductor interfaces a photovoltage *U*_PV_ may develop and thus also at the current collector|STO interface. In equilibrium, we can expect an electrostatic potential step at this interface, *i.e.* a certain space charge potential, and illumination of STO together with diffusion of photo-generated charge carriers along this interface modifies this space charge. Thus, a kind of Schottky solar cell with photovoltage *U*_PV_ results. Its initial value is quantified by *U*_PV,max_ and is found shortly after switching on UV-light. Owing to the slight p-type conductivity of nominally undoped STO,^19,20^ a negative polarity is expected at the metal, in accordance with the measurements. Hence, *U*_batt_ and *U*_PV_ show different polarity and partly cancel each other under illumination.

A quantitative understanding of *U*_PV_, however, is rather challenging, since the space charge at the STO/current collector interface is most probably also strongly affected by oxygen vacancies and their chemical potential difference between interface and bulk. Since oxygen vacancies in slightly acceptor-doped (and thus also in nominally undoped) STO are usually in majority compared to electronic charge carriers, holes may only follow the space charge profile given by the oxygen vacancy depletion.^24^ Moreover, the interface is not fully illuminated in our case and lateral charge carrier diffusion with inhomogeneous quasi Fermi levels may play a role.

Without existence of any UV induced stoichiometry change, *i.e.* without any *U*_batt_, this photovoltage could be used as the driving force of a current in our electrochemical cell; it acts like a voltage source located at the current collector STO interface, see Fig. 5. Short-circuiting of current collector and counter electrode (bottom) would thus cause electrochemical oxygen reduction at the bottom side of the cell and oxygen release at its top side. A similar coupling of photovoltage into a solid state electrochemical cell was used in ref. 11 to pump oxygen from low to high pressures. In our study this operation mode was not further investigated.

Please note that existence of *U*_PV_ is not at all a requirement for the build-up of *U*_batt_; *U*_batt_ and *U*_PV_ refer to very different processes. However, the additional oxygen incorporation under UV light changes all defect concentrations with time, therefore also the space charge at the current collector|STO interface. Accordingly, *U*_PV_ exhibits a time dependence. In our case, the lowering of the oxygen vacancy concentration by UV might reduce the corresponding space charge potential under UV and thus also *U*_PV_. This is indicated by the time-dependent *U*_PV_(*t*) (green line) in Fig. 5. Adding the two contributions, *U*_PV_ and *U*_batt_, yields the measureable black sum voltage curve in Fig. 5.

## 4. Measurements for validating the interpretation

### 4.1. Dependence on the current collector material

**Fig. 6:**
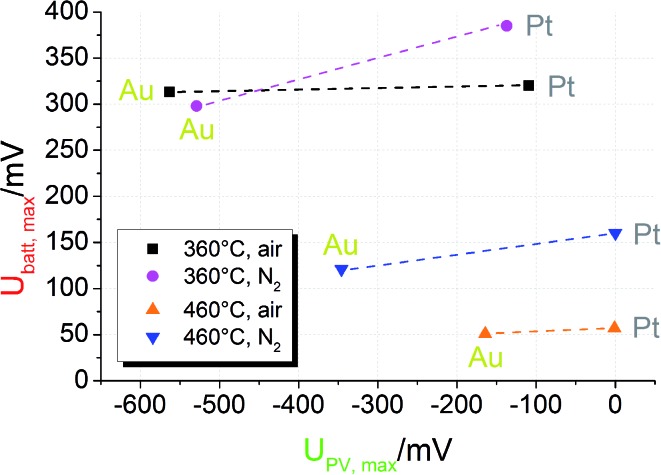
Plot of *U*_PV,max_ *vs. U*_batt,max_ for STO based SOPECs of series one. The lines connect data points recorded at the same temperature and under the same gas atmosphere. Under the same conditions *U*_PV,max_ strongly changes when the current collector material is changed. *U*_batt,max_, however, changes only slightly. This indicates not only that the PV voltage is created at the current collector|STO interface but also that two distinct processes contribute to the total voltage.

To further illustrate the presence of the two voltage contributions mentioned above, we first compare *U*_batt,max_ and *U*_PV,max_ for different STO samples of series one. At the time when *U*_PV,max_ is reached, *U*_batt_ has only been built up to a negligible extent. Shortly after UV exposure *U*_batt,max_ is still present, but *U*_PV_ is already gone. Therefore, a separate analysis should be possible. The comparison of *U*_batt,max_ and *U*_PV,max_ for samples with Pt and Au current collector in different measurement conditions is shown in Fig. 6; the lines indicate experiments performed at the same temperature and under the same gas atmosphere but with different current collector materials.

First, the photovoltaic voltage clearly depends on the current collector material, with Au leading to much larger *U*_PV,max_ values. Second, *U*_batt,max_ remains almost constant when the current collector is changed. Both observations are in accordance with our model: since *U*_PV_ originates from the current collector|STO interface, it has to depend on the current collector material. In Schottky's model of space charges at metal/semiconductor interfaces, for example, a linear relation between the barrier height and the work function difference is expected. Hence, also the photovoltage has to depend on the current collector work function. Even though the different work functions of Pt and Au may partly account for the measured difference of *U*_PV_, complications of Schottky's model are expected due to electronic interface states^25^ and due to oxygen vacancies also affecting the space charges at STO interfaces.^24^

*U*_batt_, on the other hand, reflects the enhanced oxygen content in the STO bulk and thus should show little or no dependence on the current collector material. Only if the current collector takes part in the oxygen exchange process of STO, an effect on *U*_batt_ may exist. A detailed interpretation of the temperature and gas dependence of the two voltages shown in Fig. 6 is beyond the scope of this paper. Here, we only emphasize that the battery-type voltage becomes smaller at higher temperatures and is larger for lower oxygen content (N_2_ with a few ppm O_2_) at 460 °C. Possibly the higher oxygen vacancy concentration expected for lower oxygen partial pressures enables larger effects by UV induced oxygen incorporation.

**Fig. 7:**
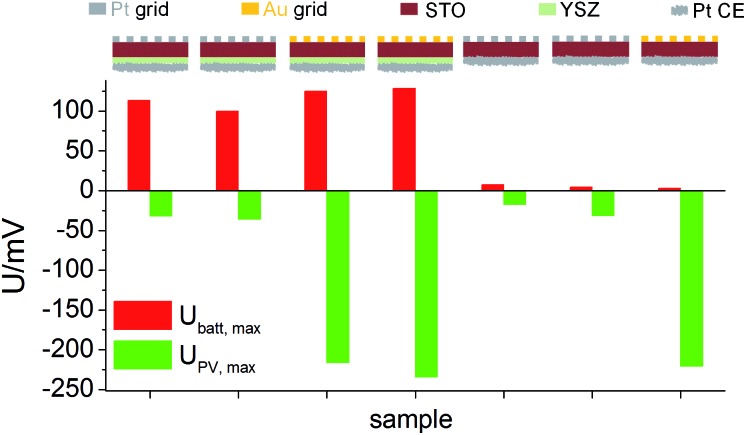
*U*_batt,max_ and *U*_PV,max_ for STO samples (series 2) with and without YSZ, measured with different current collector metals in air at 360 °C. *U*_batt,max_ depends on the presence of YSZ, but not on the current collector material. *U*_PV,max_ depends on the current collector material, but not on the presence of YSZ.

Similar experiments were performed for the second series of STO based SOPECs. In that case, also STO samples without YSZ electrolyte were investigated. The results are displayed in a bar chart in Fig. 7. *U*_batt,max_ was similar for STO samples containing YSZ (entire SOPECs), no matter which current collector material was used. When the sample had no YSZ electrolyte, *U*_batt,max_ was very small compared to the samples with YSZ, *e.g.* 7.3 mV for a sample without YSZ (Pt grid) and 114 mV for a complete SOPEC with Pt grid. Conversely, the voltage *U*_PV,max_ was similar for samples having the same current collector material, irrespective of whether or not the sample had an electrolyte layer. |*U*_PV,max_| was more than 200 mV when Au was used, but less than 40 mV for STO with Pt grid.

As for the first STO sample series, the results indicate that *U*_batt_ and *U*_PV_ are caused by two different processes and that *U*_PV_ is generated at the interface between current collector and STO. The strong enhancement of *U*_batt,max_ by the introduction of the electrolyte layer into the cell geometry further supports the interpretation of this voltage contribution as a Nernst-type voltage, originating from the different chemical potentials of oxygen in STO and the atmosphere at the counter electrode. The still existing small *U*_batt,max_ value of a few mV found in the absence of the YSZ electrolyte might be a consequence of the one-sided oxygen incorporation into STO due to asymmetric illumination. While the systematic dependencies of *U*_PV,max_ and *U*_batt,max_ are qualitatively the same in both STO sample series, the absolute values of both voltages were smaller in sample series two, despite the same measurement conditions. This difference may be caused by variations in purity of the STO single crystals of different batches but was not further investigated in this study.

### 4.2. Dependence of voltages on UV illumination time

**Fig. 8:**
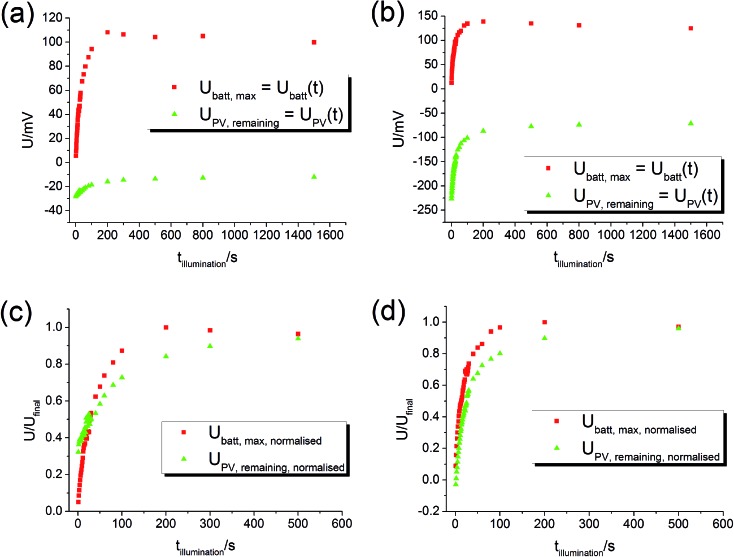
Plots of *U*_batt,max_ and *U*_PV,remaining_ as a function of illumination time at 360 °C in air. Since these values represent *U*_batt_ and *U*_PV_ at time *t* = *t*_illumination_, the diagrams can be interpreted as plots of *U*_batt_(*t*) and *U*_PV_(*t*). (a) Sample from series two with Pt current collector. (b) Sample from series two with Au current collector. (c) and (d) Normalised curves of (a) and (b). The time evolution of *U*_PV_ and *U*_batt_ is comparable.

By varying the UV illumination time it is possible to track the evolution of *U*_PV_ and *U*_batt_ as a function of time. After a certain illumination time *t*_illumination_ the voltage *U*_batt,max_, measured after switching off the UV light, corresponds to *U*_batt_ of an experiment with still ongoing illumination. Hence, the true *U*_batt_ curve of a long-term illumination (*cf.* Fig. 5) can be reconstructed from many experiments with smaller illumination times. Similarly, the PV voltage present after a certain time of illumination can be used to reconstruct the true *U*_PV_ curve. This PV voltage *U*_PV,remaining_ is determined from the fast voltage jump found after switching off the light. The graphs in Fig. 8 show the measured *U*_batt,max_ and *U*_PV,remaining_ for different illumination times – these plots can be considered as curves of *U*_batt_(*t*) and *U*_PV_(*t*).

The battery voltage *U*_batt_ starts almost at zero and rises continuously until it becomes rather constant. The maximum value in the 100 mV range is very similar for both current collector materials (Pt, Au). The PV voltage *U*_PV_ starts approximately at the maximum PV voltage, which was about –35 mV for this STO sample with Pt current collector and –220 mV for the sample with Au current collector. Then, |*U*_PV_| decreases. The normalised curves (Fig. 8(c) and (d)) show that the time scales of the *U*_batt_ and *U*_PV_ changes are similar, in accordance with our interpretation of both changes originating from the oxygen incorporation into STO under UV illumination. Hence, all these results are in very good agreement with our model and the time-dependent voltage contributions sketched in Fig. 5.

### 4.3. Dependence of voltages on equilibration time

For an electrochemical solid oxide cell with identical oxygen partial pressures at both electrodes Nernst's equation predicts zero cell voltage. In contrast to this, a voltage offset *U*_dark_ partly larger than 100 mV was measured on STO samples with YSZ layer even without UV illumination. On STO samples without YSZ layer, however, *U*_dark_ was in the order of 0.1 mV and thus negligible. The YSZ electrolyte being necessary for a significant *U*_dark_ suggests that it is of electrochemical origin. The *U*(*t*) curves showed that several hours after switching off UV the voltage had again reached its original value – *U*_dark_ and we therefore conclude that the oxygen exchange reaction is in equilibrium after several hours. Hence, *U*_dark_ is most probably not caused by a non-equilibrium value of the oxygen chemical potential in STO.

**Fig. 9:**
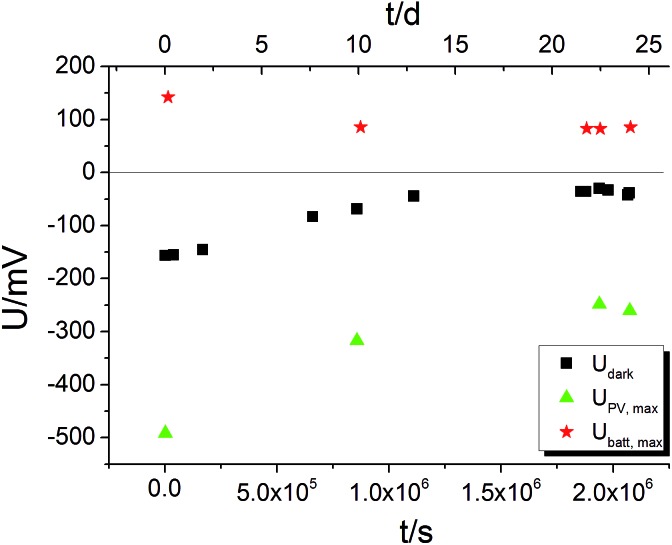
Long-term *U*(*t*) measurement on a STO based SOPEC (series two, Au grid) at 360 °C. All three voltages decrease in magnitude within three weeks. *U*_batt,max_ and *U*_PV,max_ were probed by short UV illumination periods (1 to 4 h each); *U*_dark_ was not affected by these short illuminations; the sample returned to the *U*_dark_ values measured before illumination within a few thousand seconds.

The time dependence of *U*_dark_ was analysed in a long-term voltage measurement on a STO sample from series two (Pt grid) exposed to 360 °C for more than three weeks with some short UV irradiation periods to follow *U*_batt,max_ and *U*_PV,max_ on this time scale. Within three weeks |*U*_dark_| decreased from its initial value of approximately 150 mV to 30–40 mV (black squares in Fig. 9). Therefore, we suggest that *U*_dark_ is at least partly related to another, yet unknown redox process which equilibrates on a long time scale, meaning that *U*_dark_ includes a kind of mixed potential effect. Also additional phenomena such as a thermovoltage could be present.^26^ Interestingly, also *U*_PV,max_ and *U*_batt,max_ decreased in magnitude during three weeks, but a substantial *U*_batt_ is still present at the end of the long-term measurement, despite strongly decreased *U*_dark_. Hence, *U*_batt_ does not rely on a non-equilibrated state of our STO based SOPECs.

### 4.4. Time-resolved impedance measurements

**Fig. 10:**
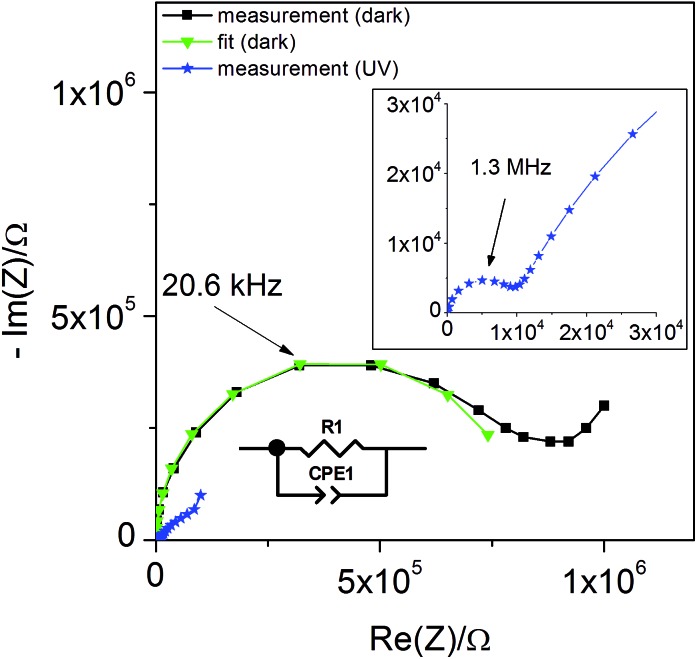
Typical impedance spectrum of a STO-based SOPEC (Au current collector, series one) at 360 °C in air. Only the high-frequency semicircle was evaluated and interpreted as the STO bulk.

Additional information on stoichiometry changes of STO upon UV illumination were gained from time-resolved electrochemical impedance measurements, carried out on a STO based SOPEC from series one with Au current collector. A typical impedance spectrum is shown in Fig. 10. Only the high-frequency semicircle was evaluated. An *R*||CPE (CPE = constant phase element, impedance *Z*_CPE_ = *Q* ^–1^(i*ω*)^–*n*^, *ω* = angular frequency) equivalent circuit was fitted to the data. The capacitance was extracted from the CPE parameters *Q* and *n* using the formula given in ref. 27:1
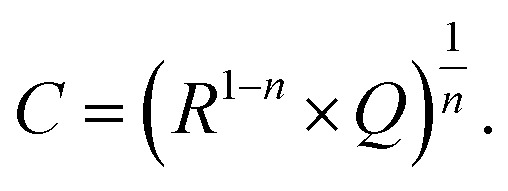



The symbols *C* and *R* denote the capacitance and resistance of the corresponding process, respectively. The relative dielectric constant *ε*_r_ was calculated assuming a parallel plate capacitor. For 360 and 460 °C, *ε*_r_ values of 218 and 204, respectively, were obtained. The compilation of literature values given in ref. 28 reports relative dielectric constants of STO between *ε*_r_ = 160 and 170 for 360 °C and between *ε*_r_ = 130 and 170 for 460 °C, which are in reasonable agreement with the values measured in the present study. The resistance expected for the YSZ thin film (a few Ω, estimated from YSZ conductivity data in ref. 29) is negligible compared to *R*_STO_ and thus cannot be resolved from the spectrum. Hence, the semi-circular high frequency impedance feature is assigned to the STO bulk, meaning that *C* = *C*_STO_ and *R* = *R*_STO_.

**Fig. 11:**
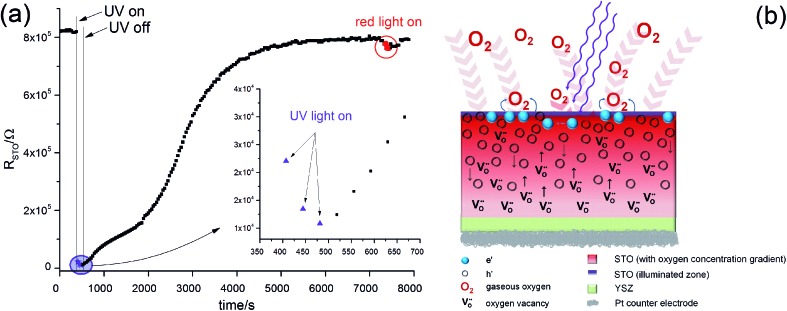
(a) Plot of *R*_STO_ extracted from impedance spectra of a STO sample with YSZ (Au current collector) as a function of time during switching UV light on and off (360 °C, air). Upon switching the light on, *R*_STO_ drops quickly by almost two orders of magnitude. After switching the light off, *R*_STO_ recovers only slowly. This phenomenon is interpreted as a consequence of oxygen incorporation during UV illumination, sketched in (b): even though UV absorption takes place only in a thin layer of the STO single crystal, chemical diffusion leads to a filling of the entire STO crystal.

In Fig. 11(a) the values of *R*_STO_ at 360 °C in air are plotted as a function of time, while UV light was switched on and off. Please note that the time resolution of this plot is limited by the time to record an impedance spectrum (*ca.* 6 to 40 seconds). When UV light was switched on, a strong drop in the SrTiO_3–*δ*_ resistance occurred within a few seconds. A corresponding impedance spectrum is shown in Fig. 10 and demonstrates that the STO arc decreases drastically. When UV light was switched off, the first measured resistance value was only slightly increased compared to *R*_STO_ under UV light. It took a few 1000 s to return to the initial resistance value.

These *R*_STO_(*t*) characteristics may depend on two effects. First, photoconductivity is expected to occur. This should show a very fast response for both switching on UV light or switching it off. In the measurement, however, we see a moderately fast jump upon switching on the light and a very sluggish relaxation of *R*_STO_, without a clear jump, after switching it off. Therefore, enhanced photoconductivity cannot explain these measurements. Most probably UV absorption takes place within a few μm (ref. 9) and diffusion lengths of the charge carriers are much smaller than the sample thickness of 0.5 mm. Therefore, any photoconductive layer exists only near to the surface and would hardly affect the total resistance measured across the entire STO single crystal.

Second, the uptake of oxygen by STO not only produces the Nernst-type voltage described above but also changes the conductivity. During oxygen incorporation, electrons are consumed and thus holes are generated according to2
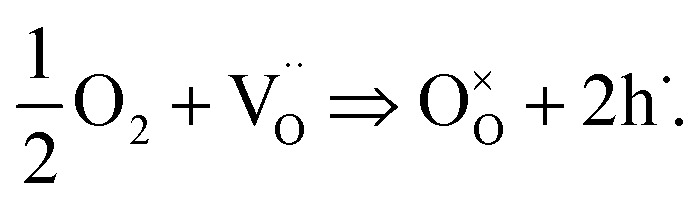



These electron holes increase the electronic conductivity. Essentially, the slight acceptor dopant present in any nominally undoped STO^18,19^ is partly compensated by oxygen vacancies and partly by electron holes. UV light lowers the vacancy concentration by oxygen incorporation and thus enhances the fraction of hole compensation. However, while photoconduction is limited to the UV absorbing region (with some extension due to diffusion of electronic charge carriers), the stoichiometry change takes place in the entire STO crystal: the enhanced oxygen chemical potential in the region with UV absorption pumps oxygen also into the remaining STO, see sketch in Fig. 11(b).

This can explain the observed *R*_STO_(*t*) behaviour. After switching on UV light the oxygen incorporation starts and takes place at a rather high rate.^12^ Provided that chemical diffusion is sufficiently fast, a comparatively quick drop of the resistance in the entire STO crystal results. Immediately after illumination, this modified stoichiometry with much more holes is still present and thus the electronic conductivity is still high. Since oxygen is released only very slowly after switching off the light, the resistance also rises very slowly towards its starting value.

Accordingly, the build-up of the battery-type voltage *U*_batt_ and the conductivity change are both caused by the increase of the oxygen content under UV light. This is also seen when comparing the two time constants, *τ*_batt_ for building up the steady-state voltage in a *U*(*t*) measurement and *τ*_*R*_STO__ reflecting the resistance decrease in the *R*_STO_(*t*) measurement. The estimation of *τ*_batt_ and *τ*_*R*_STO__ was done by evaluating the total change in voltage or resistance and searching the time where it had a value of *R*_STO_/*e* or (1 – *e*) × *U*. Furthermore, the time constants of oxygen chemical diffusion with diffusion coefficient *D*_chem_ were estimated from literature data.^21^ It was assumed that trapping of holes is not important; then values of 1 × 10^–4^ cm^2^ s^–1^ (360 °C, air) and 2.5 × 10^–4^ cm^2^ s^–1^ (460 °C, air) result and those were converted into time constants *τ*_lit_ = *d* ^2^/*D*_chem_ using the sample thickness *d*. The results of these estimations are summarized in Table 1. All time constants are in reasonable agreement, which supports the assumption that both the STO resistance decrease and the voltage *U*_batt_ are caused by incorporation of oxygen into the STO bulk, driven by UV light.

**Table 1:** Comparison of estimated time constants for the voltage changes in *U*(*t*) measurements under UV light (*τ*_batt_) and the drop of resistance in *R*_STO_(*t*) measurement (*τ*_*R*_STO__), performed on STO samples with YSZ (series one). Also estimates of time constants from chemical diffusion coefficients in air are given (*τ*_lit_)

Current collector material	Temperature/°C	Atmosphere	*τ*_batt_/s	*τ*_*R*_STO__/s	*τ*_lit_/s
Pt	360	Air	6	—	25
Pt	460	Air	20	—	10
Pt	360	N_2_	10	—	—
Pt	460	N_2_	4	—	—
Au	360	Air	13	24	25
Au	460	Air	4	8	10
Au	360	N_2_	18	70	—
Au	460	N_2_	16	13	—

Also a further data analysis supports the plausibility of our interpretations: the conductivity of STO after UV light reflects a STO single crystal with very high chemical potential of oxygen, generated by UV-driven oxygen uptake. By using the defect chemical data for Fe-doped STO given in ref. 18, the change in *R*_STO_ (or *σ*_STO_) due to UV light can be used to calculate a corresponding change in oxygen chemical potential. The “equivalent *p*(O_2_) change” is the change in oxygen partial pressure that would be necessary to effect the same hole concentration change and thus conductivity change as the oxygen incorporation by UV light. This can in turn be used to calculate the expected voltage change *U*_batt,max_ *via* Nernst's equation. The dopant content of our nominally undoped STO is needed for these calculations and it was estimated such that the defect chemical data set of ref. 18 reproduced conductivity values measured at 550 °C, *i.e.* at a temperature where equilibration with the gas atmosphere is ensured. An acceptor concentration of 5 × 10^17^ cm^–3^ could be estimated. The conductivity ratio 
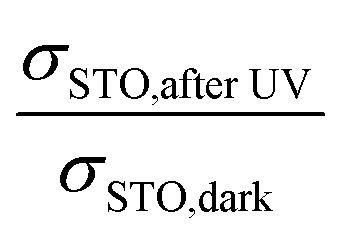
 used for calculating an estimate of *U*_batt,max_ requires a (hypothetical) dark conductivity of an equilibrated sample. Since in the dark equilibration is very sluggish, this value was determined from the estimated acceptor dopant level (see above) and the defect chemical data in ref. 18. The results of these calculations and the measured values of *U*_batt,max_ are presented in Table 2. They are in very reasonable agreement, especially at lower temperature, which again supports the consistency of our model.

**Table 2:** Calculation of *U*_batt,max_ from the change in *R*_STO_ (and thus in conductivity *σ*_STO_) during illumination with UV light (SOPEC from series one with Au current collector), compared to the measured maximum battery voltages. The partial pressure change Δlog *p*(O_2_) can reproduce the conductivity change within the framework of the defect chemical data set of STO in ref. 18

Temperature *T*/°C	Atmosphere	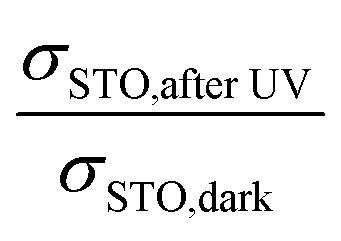	Δlog *p*(O_2_)	*U*_batt,max_/mV calc. from *R*_STO_	*U*_batt,max_/mV measurement
360	Air	19.2	7.2	219	313
460	Air	6.7	4.5	157	51
560	Air	1.5	8.8	35	7
360	N_2_	92.2	1.0	311	298
460	N_2_	22.1	6.8	237	121

### 4.5. Current measurements

**Fig. 12:**
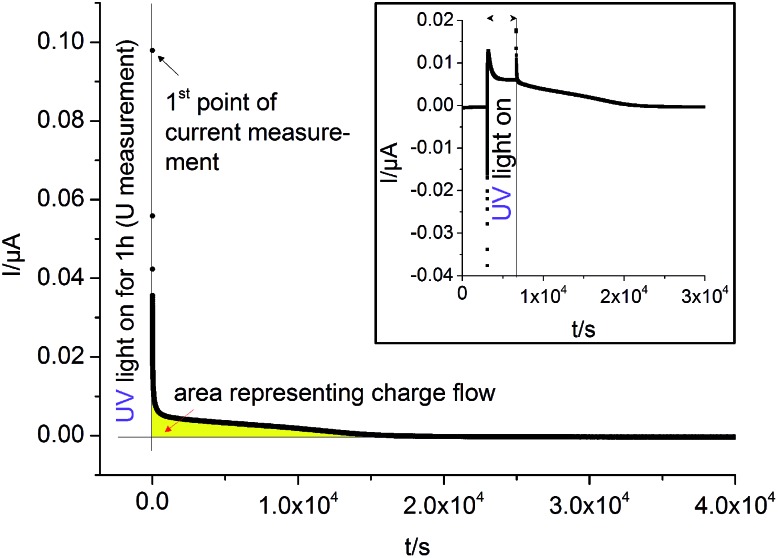
Typical *I*(*t*) curves recorded on STO samples with YSZ electrolyte (series two, Pt current collector) at 360 °C in air. The large diagram shows the short circuit current measured in dark after “charging” STO by UV light under open circuit conditions. We assume that after switching off the UV light and connecting an amperemeter, oxygen is almost exclusively released *via* the counter electrode. Then, the amount of oxygen initially taken up by STO can be estimated from the area under the *I*(*t*) curve (yellow) and Faraday's law. The inset shows the short circuit current *I*(*t*) of such a SOPEC under illumination (*i.e.* in oxygen pumping mode) and after illumination (battery mode).

Current measurements were carried out on STO samples (series 2) with and without YSZ electrolyte. First, UV light was switched on for 1 hour while the open circuit voltage was measured. Then, light was turned off and after 10 s a relay switched the connection from the voltmeter to an amperemeter. A typical curve is displayed in Fig. 12. By illuminating SOPECs under open circuit conditions, the STO electrode gets filled with oxygen. When the amperemeter is connected, the cell becomes short-circuited and the excess oxygen can not only leave the STO electrode *via* the surface but also by moving through the electrolyte to be released at the counter electrode. The electrons which are then liberated at the counter electrode are transported to the STO electrode in the outer circuit and charge balance the oxide ion motion.

Assuming that oxygen release *via* the surface is negligibly slow compared to oxygen release *via* the counter electrode, the amount of oxygen incorporated under UV, Δ*n*_O_ [mol], can be calculated from the oxygen release current, *I*(*t*), using Faraday's law:3
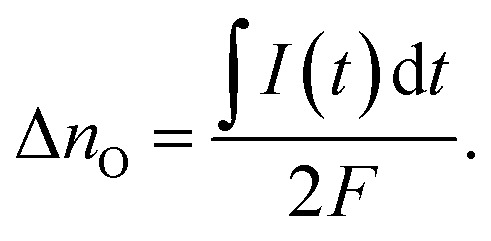



The symbol *F* represents Faraday's constant. Fig. 12(a) shows a typical *I*(*t*) curve. The change in the amount of oxygen atoms is the same as the change in the amount of oxygen vacancies, but with opposite sign: 
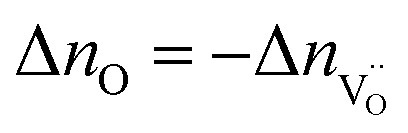
. The relative change in the amount of oxygen vacancies corresponds to the relative change of the non-stoichiometry *δ*,4
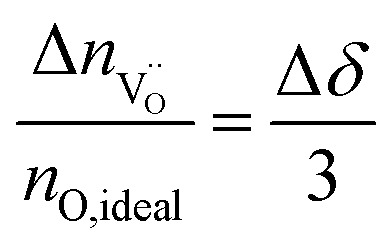



Thus we can compute the change in non-stoichiometry Δ*δ* from the *I*(*t*) curve. The amount of oxygen contained in a perfect STO crystal, *n*_O,ideal_ was determined from the sample volume, the density and the molar mass of STO.^30^

Furthermore, by using the defect chemical data available for weakly Fe-doped STO,^18^ we can compute the oxygen partial pressure that would have been necessary to effect the same change in concentration of oxygen vacancies. From the ratio between this pressure and the ambient oxygen partial pressure we can again calculate the maximum battery voltage *U*_batt,max_ expected across the electrolyte according to Nernst's equation:5
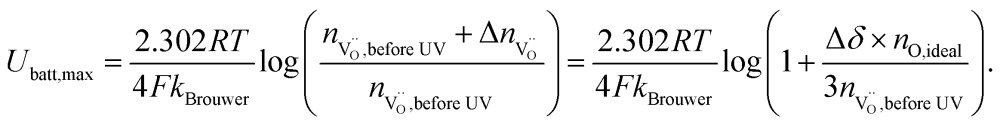



The symbol 
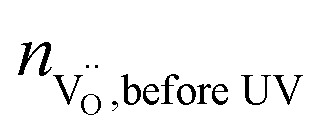
 represents the “native” amount of oxygen vacancies (before UV light was switched on). It was determined from the defect model in literature^18^ and the estimated acceptor concentration (5 × 10^17^ cm^–3^, see above); *k*_Brouwer_ denotes the slope of the oxygen vacancy concentration in the Brouwer diagram and was obtained from defect chemical modelling again based on literature data^18^ (*k*_Brouwer_ = –0.16).

The change in oxygen non-stoichiometry Δ*δ* calculated from the measured currents for four different samples of STO series two (two with Pt, two with Au) is between –1.4 × 10^–6^ and –3.5 × 10^–6^. The corresponding average of Δ*δ* ± standard deviation of the average (–2.3 × 10^–6^ ± 0.4 × 10^–6^) can be compared with the maximum possible stoichiometry change, Δ*δ*_max_, resulting from the estimated amount of oxygen vacancies present before illumination. We find Δ*δ*_max_ = –3.1 × 10^–6^ and can thus conclude that a substantial fraction of the existing oxygen vacancies becomes filled upon illumination with UV light. From the measured average stoichiometry change and eqn (5) we can calculate a battery voltage *U*_batt,max_ and find 90 ± 22 mV which is in good agreement with the voltages measured for the same samples (117 mV ± 27 mV).

### 4.6. Similar results on TiO_2_ samples

**Fig. 13:**
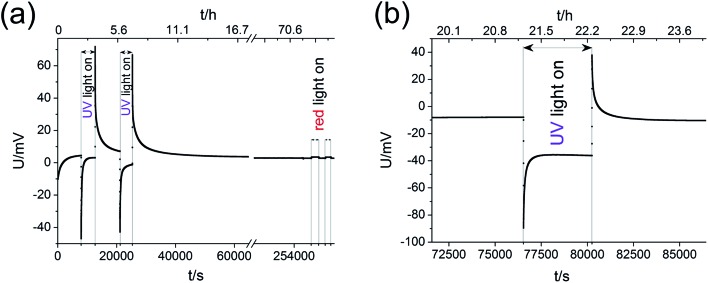
*U*(*t*) curves for two different samples with TiO_2_ thin film microelectrode (460 °C, air). Their shapes are similar to the curves measured on STO samples. In (a) also the effect of red light was probed and it was found to be negligible. (b) highlights the processes caused by a single UV illumination phase.

The shapes of the *U*(*t*) curves measured on SOPECs with TiO_2_ thin films are similar to those measured on the STO samples, see Fig. 13 (460 °C, air). Here, almost no voltage offset was present. As on STO, red light caused almost no effect. These measurements are interpreted in analogy to the results on STO and we therefore conclude that TiO_2_ also incorporates oxygen upon illumination with UV light. The corresponding battery voltages *U*_batt,max_ were in the range of 30–70 mV at 460 °C in air and thus in a similar range as for STO. The voltage *U*_PV,max_ was 40–80 mV. The presence of the same effect for two very different electrodes (different material, single crystal *vs.* polycrystalline thin film) indicates the general relevance of the observed effects. More details on the high-temperature photoelectrochemical properties of TiO_2_ are given in ref. 26.

### 4.7. Outlook

The measurements revealed that UV light affects the thermodynamic properties of mixed conducting oxide electrodes and that SOPECs can be realized. In principle, two operation modes of such illuminated SOPECs are conceivable: one mode was already discussed in Section 4.5 with charging of the oxide working electrode by light and discharging it after illumination *via* an external load. This operation mode resembles a battery and we may refer to it as a kind of “light charged oxygen battery”. Here, the chemical release of oxygen *via* the surfaces of the working electrode is a competing process and can be considered as self-discharging. Certainly, the short circuit currents achieved so far are much too low for any application. The same is true for the amount of stored charge since only oxygen vacancy concentrations in the ppm range were affected so far. However, further research activities may identify optimized mixed conducting oxides that enable higher ionic and electronic conductivities and large stoichiometry changes upon illumination, preferably with light absorption in the visible range.

A second operation mode is based on an external short circuit during continuous illumination. Then, oxygen is continuously incorporated into the working electrode and pumped through the electrolyte to the counter electrode. This mode would also allow pumping against an external oxygen partial pressure difference and may thus be considered as the solid oxide equivalent of water based photoelectrochemical cells. Operating our cell in this oxygen pumping mode is indeed possible. For Pt current collectors with small photovoltages, currents in the range of several nA were found, see inset in Fig. 12. The short circuit current in the oxygen pumping mode was similar to that found in the battery mode after illumination. This similarity is not surprising, provided oxygen incorporation into STO (required in the oxygen pumping mode but not in the battery mode) is fast under UV. Then the current in both modes is limited by oxygen diffusion through STO and oxygen release at the counter electrode. However, the current in the pumping mode becomes rather constant after some time while the discharge current in the battery mode decreases. Please note that the photovoltage originating from the current collector/oxide interface can hamper the oxygen pumping mode due to its opposite polarity; in our specific case of Au current collectors the remaining overall driving force for oxygen pumping was even very small, and hence also the short circuit pumping current. An ohmic contact at the current collector/oxide interface, without significant *U*_PV_, would be beneficial for this mode. Moreover, it could be highly attractive to operate such a cell under reducing conditions (H_2_/H_2_O). Ultimately, also solid electrolyte based photoelectrochemical water splitting might be realized in this manner.

## 5. Conclusions

In this study time-resolved voltage, impedance and current measurements were performed on solid oxide electrochemical cells with a STO working electrode while switching on and off UV light. Typical voltage–time curves showed a fast jump to more negative voltages when UV light was switched on, followed by an opposite change to a plateau. Upon switching UV light off, there was a jump to positive voltages and only slowly the starting value was reached again. The STO resistance, monitored by impedance spectroscopy, showed a rather sharp decrease after UV light was turned on, but a very slow re-increase after switching off UV. Short circuit currents after UV illumination revealed that a charge was accumulated upon illumination under open circuit conditions.

All these measurements can be consistently explained by assuming that apart from a photovoltaic effect UV light causes oxygen incorporation into STO and thus a stoichiometry change. In an electrochemical cell this leads to a Nernst-type (battery) voltage. The evolution of this battery voltage with time could be reconstructed from measurements with varying illumination time. The decrease in STO resistance reflects the fact that oxygen incorporation needs electrons, so hole concentrations are increased and enhance the electronic conductivity. The measured resistance changes can be converted into oxygen partial pressure and voltage changes using defect chemical data from literature and the resulting voltages are in reasonable agreement with the measured battery voltage. The different kinetics of voltage and resistance change under UV illumination and in darkness indicate that UV light accelerates oxygen incorporation but not oxygen release.

The amount of oxygen incorporated during illumination (equivalent to the capacity of this “light charged battery”) could be estimated from the discharge current measurements by assuming that oxygen release *via* the surface is negligibly slow. According to this calculation most oxygen vacancies present in STO are filled during UV illumination. This UV-driven oxygen storage is not restricted to STO; the same effects were also found for TiO_2_ thin films. Illuminating a cell under short circuit conditions enables continuous operation of a solid oxide photoelectrochemical cell (SOPEC). Future research has to reveal whether also much larger stoichiometry changes and larger currents can be achieved in such SOPECs.
